# Peripheral Nerve Regeneration Using a Cytokine Cocktail Secreted by Skeletal Muscle-Derived Stem Cells in a Mouse Model

**DOI:** 10.3390/jcm10040824

**Published:** 2021-02-17

**Authors:** Daisuke Maki, Tetsuro Tamaki, Tsuyoshi Fukuzawa, Toshiharu Natsume, Ippei Yamato, Yoshiyasu Uchiyama, Kosuke Saito, Kenji Okami

**Affiliations:** 1 Department of Otolaryngology, Tokai University School of Medicine, 143 Shimokasuya, Isehara, Kanagawa 259-1193, Japan; d.maki@tokai.ac.jp (D.M.); kousukesaitou427@yahoo.co.jp (K.S.); okami@is.icc.u-tokai.ac.jp (K.O.); 2 Muscle Physiology and Cell Biology Unit, Tokai University School of Medicine, 143 Shimokasuya, Isehara, Kanagawa 259-1193, Japan; fukuzawa-tsuyoshi@tokai-u.jp (T.F.); nt554270@tsc.u-tokai.ac.jp (T.N.); ippei-y@is.icc.u-tokai.ac.jp (I.Y.); y-uchi@is.icc.u-tokai.ac.jp (Y.U.); 3 Department of Physiology, Tokai University School of Medicine, 143 Shimokasuya, Isehara, Kanagawa 259-1193, Japan; 4 Department of Radiation Oncology, Tokai University School of Medicine, 143 Shimokasuya, Isehara, Kanagawa 259-1193, Japan; 5 Department of Medical Education, Tokai University School of Medicine, 143 Shimokasuya, Isehara, Kanagawa 259-1193, Japan; 6 Department of Orthopedic Surgery, Tokai University School of Medicine, 143 Shimokasuya, Isehara, Kanagawa 259-1193, Japan

**Keywords:** severe peripheral nerve injury, regenerative medicine, therapeutic agent, therapeutic adjunct, facilitation effect

## Abstract

Severe peripheral nerve injury, which does not promise natural healing, inevitably requires clinical treatment. Here, we demonstrated the facilitation effect of peripheral nerve regeneration using a cytokine cocktail secreted by skeletal muscle-derived stem cells (Sk-MSCs). Mouse sciatic nerve was transected with a 6 mm gap and bridged collagen tube, and the culture supernatant of Sk-MSCs with 20% adult mouse serum (AMS)/Iscove’s modified Dulbecco’s medium (IMDM) was administered into the tube immediately after the operation, followed by an injection once a week for six weeks through the skin to the surrounding tube of the cytokine (CT) group. Similarly, 20% AMS/IMDM without cytokines was administered to the non-cytokine control (NT) group. Tension recovery in the plantar flexor muscles via electrical stimulation at the upper portion of the damaged nerve site, as well as the numerical recovery of axons and myelinated fibers at the damaged site, were evaluated as an index of nerve regeneration. Specific cytokines secreted by Sk-MSCs were compared with damaged sciatic nerve-derived cytokines. Six weeks after operation, significantly higher tension output and numerical recovery of the axon and myelinated fibers were consistently observed in the CT group, showing that the present cytokine cocktail may be a useful nerve regeneration acceleration agent. We also determined 17 candidate factors, which are likely included in the cocktail.

## 1. Introduction

Peripheral nerve injury is frequently caused by various traumatic injuries such as penetrations, crushes, tractions, and lacerations, following various sports events and road accidents [1,2]. In severe cases, nerve injury with a long gap is problematic and does not promise natural healing; thus, clinical treatment is necessary. In such cases, nerve autograft therapy has been attempted as the current surgical standard to improve recovery [3,4]. However, the results are likely unstable and insufficient despite the sacrifice of healthy nerves and their related functions. Therefore, various alternative methods have been developed, such as bridging the nerve gap with a tube of some type of scaffold, or a combination of scaffold bridging and stem cell transplantation. Through these studies, it appears that scaffold bridging combined with stem cell transplantation consistently achieves better results than scaffold bridging alone [4]. Various cell sources have been used for nerve regeneration, such as Schwann cells and/or Schwann-like cells induced from cultivated bone marrow stromal cells [5], olfactory ensheathing cells [6], adipose tissue-derived cells [7], and skeletal muscle-derived multipotent stem cells (Sk-MSCs) [8]. At present, Sk-MSC transplantation demonstrates maximum effects, with a >80% average of numerical (axon and myelinated fibers) and functional recovery (tetanic tension output via electrical stimulation) following active cell engraftment and differentiation of Schwann cells, perineurial/endoneurial cells associated with vascular endothelial cells, and pericytes [8]. 

The first and primary purpose of stem cell transplantation therapy is the differentiation and incorporation of engrafted cells, which exerts a facilitation effect on the axonal extension/reconnection following the formation of the Büngner band and perineurium/endoneurium [8]. However, it has been found that paracrine effects of transplanted cells are also important and should be considered a secondary purpose [9,10,11]. In fact, in human Sk-MSC transplantation experiments, it has been suggested that the average total/final numerical/functional recovery (75–100%) over 12 weeks may include an approximately 60–80% contribution of the paracrine effects [11]. This result demonstrates the possibility that the paracrine effects of Sk-MSCs may be used as a nerve regeneration acceleration agent. Using the same concept, a tissue regeneration accelerating agent has also been reported using platelet-rich plasma (PRP) injections [12,13]. 

In the present study, we investigated whether the peripheral nerve regeneration in vivo, such as axonal extension/reconnection, could be facilitated by repetitive administration of the mouse Sk-MSC-derived cytokines. Therefore, this study is the first step in regenerative medicine without the use of stem cells, but with the use of stem cell functions, with the aim of achieving broader and easier therapeutic utility. In other words, this is stem cell therapy without cell transplantation, but uses the administration of cytokine cocktails expressed by the stem cells. 

## 2. Experimental Section

### 2.1. Animals

Green fluorescent protein transgenic male mice (GFP-Tg mice; C57BL/6 TgN[act EGFP]Osb Y01, provided by Dr. M. Okabe, Osaka University, Osaka, Japan) [14] were used as donor mice for nerve graft transplantation (age 8–12 weeks, *n* = 6), and wild-type male female mice (C57BL/6N) were used as recipients (age 8–12 weeks, *n* = 31). All experimental procedures were approved by the Tokai University School of Medicine Committee on Animal Care and Use (No. 203034), and all experiments were performed in accordance with relevant guidelines and regulations. 

### 2.2. Isolation of Nerve Graft and Preparation of the Nerve Regeneration Assessment Model

**Figure 1:**
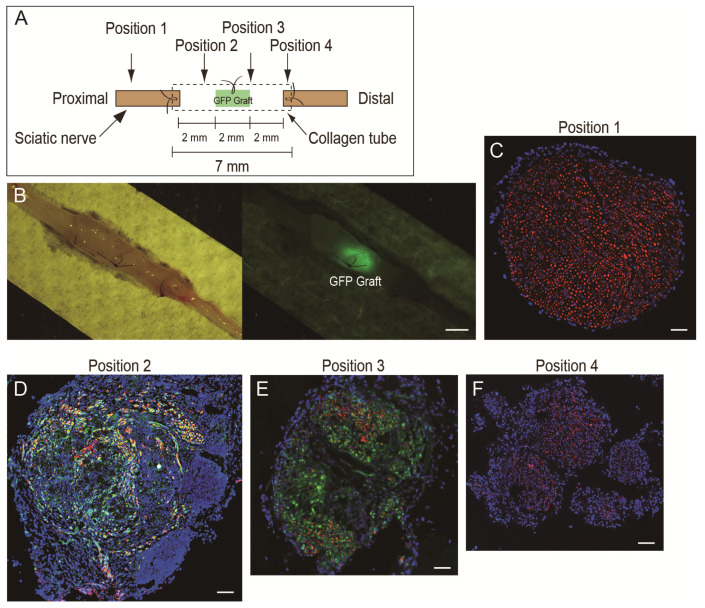
Schematic drawing of the nerve regeneration assessment model (**A**) and typical features of the regenerated bridging nerve (**B**–**F**). In (**A**), the GFP graft was a piece of sciatic nerve (2 mm) obtained from GFP-Tg mouse. The 6 mm nerve gap was made using a 7 mm collagen conduit. (**B**) Typical macroscopic view of regenerated nerve. GFP nerve graft could be seen at the center of the tube conduit under fluorescence stereomicroscopy. Scale bar = 1 mm. (**C**–**F**): Typical cross-sectional view of the regenerated nerve of the NT group at position 1–4. Sections were stained with N200 (red) as axon staining and nuclear staining with DAPI (blue). Scale bars = 100 μm. GFP-Tg, green fluorescent protein transgenic mice; DAPI, 4′,6-diamidino-2-phenylindole; NT, non-cytokine administered control group.

Nerve sampling was performed under an overdose of pentobarbital (60 mg/kg, Schering-Plough, combined with butorphanol tartrate 2 mg/kg, Meiji Seika, Tokyo, Japan, i.p.). Sciatic nerves (approximately 10 mm each) of GFP-Tg mice were removed from both side and used as the nerve graft in the nerve regeneration assessment model (Figure 1A). 

Preparation of the nerve regeneration model in wild-type mice was performed under inhalation anesthesia (isoflurane; Abbott, Tokyo, Japan). Body temperature was monitored by a rectal probe and maintained at 36 ± 1 °C with radiant heat throughout the surgical procedure. During surgery, an analgesic nonnarcotic opioid (butorphanol tartrate; 0.1 mg/kg subcutaneous infusion, Meiji Seika Pharma, Tokyo, Japan) was administered, as needed. The right sciatic nerve was transected, and an approximately 7 mm long nerve portion was removed. Thereafter, both transected stumps were bridged using a collagen tube (7 mm long, diameter 1.0 mm, Renerve, NIPRO, Osaka, Japan), and the length of the gap was adjusted to 6 mm (0.5 mm on both sides was suture marginal). At this time, a 2 mm long GFP nerve graft was stitched in the central portion of the tube (Figure 1A,B). This was used as an indicator to obtain the histological sections at position 1–4 in Figure 1A,C–F more precisely. After the operation, the surgical wound was sutured, a transparent sterile/analgesic plastic dressing (Nobecutan spray; Yoshitomi Chemical, Japan) was sprayed over the wound, and penicillin (4000 units/100 mL) was subcutaneously administered to prevent infection.

### 2.3. Preparation and Administration of the Cytokine Cocktail

The cytokine cocktail was obtained as the culture supernatant of Sk-MSCs. Thus, Sk-MSCs were isolated from the skeletal muscle of wild-type mice strictly following a previously reported method [8]. Enzymatically isolated Sk-MSCs, which were primarily composed of a mixture of reported Sk-34 (skeletal muscle-derived CD34+/45−) and Sk-DN (skeletal muscle-derived CD34−/45−) cells [15,16], were cultured in Iscove’s modified Dulbecco’s medium (IMDM) containing 20% fetal calf serum (100 units/mL penicillin G, 100 μg/mL streptomycin sulfate, 10 μg/mL gentamycin sulfate, and 0.1 mM β-mercaptoethanol) for 5 d. Thereafter, cultures were washed well, the medium changed to IMDM containing 20% wild-type adult mouse serum (AMS), and cultured for 24 h. The culture supernatant was collected as the cytokine cocktail, centrifuged at 15,000 rpm, and the supernatant was frozen and stored at −80 °C until use. 

Operated recipient mice were divided into two groups: the cytokine-treated group (CT, *n* = 10) and the non-treated control (NT, *n* = 15) group. Immediately after the bridging operation, a cytokine cocktail (20 μL) was injected into the bridged collagen tube of the CT group, and the same amount of 20% AMS/IMDM was injected into the NT group. Additionally, administration of a 0.1 mL cytokine cocktail was performed via needle injection through the skin and buttock muscles at one shot/week for 6 weeks for infusion with the cocktail around/inside the collagen tube. The NT group received the same amount of 20% AMS/IMDM. 

### 2.4. Functional Assessment of Downstream Muscles

As one of the prominent and quantitative indicators of functional recovery for long-gap sciatic nerve transection, tetanic tension outputs of the downstream lower hind limb plantar flexor muscles were measured in both the non-operated left contralateral side and the operated right side, and the recovery ratio between CT (*n* = 6) and NT (*n* = 8) groups was compared. Six weeks after the operation, measurements were performed in situ under anesthesia (isoflurane; Abbot, Osaka, Japan), and body (rectal) temperature was maintained at 36 ± 1 °C with radiant heat during the measurement. Tetanic tension output was measured as the unit of plantar flexors, together with plantaris (PLT), soleus (SOL), and gastrocnemius (GAS) muscles. The Achilles and distal tendons of the PLT muscle were exposed and tied together with a stainless-steel hook and were connected to the force transducer. Similarly, sciatic nerves (approximately 10 mm) on both sides were carefully exposed, and a stimulation electrode was situated at the 5 mm upper portion (proximal) of the bridged tube. Exposed tissues were coated with mineral oil to prevent drying out and to minimize electrical noise interference. Details for setting up the instrument and the method of tension measurement have been described previously [11,17,18]. Tetanic tension output was considered to represent the total functional recovery of nerve-muscle units, and the recovery ratio was determined based on the contralateral side.

### 2.5. Immunohistochemistry and Numerical Analysis

Following functional assessment of downstream muscles, recipient mice were administered an overdose of pentobarbital (60 mg/kg, i.p.), and the animal was exsanguinated. Operated sciatic nerves in each group were freshly removed and extended and pasted on the paper filter. Then, they were photographed, and fixed overnight in 4% paraformaldehyde/0.1 M phosphate buffer (4% PFA/PB), and washed with a graded sucrose series (0–25%)/0.01 M phosphate-buffered saline, in order to substitute a tissue fluid to prevent an ice crystal formation and tissue cracking for the histological analysis. Thereafter, nerve samples were cut at the specified portion as shown in Figure 1A (positions 1–4), embedded in optimum-compound (O.C.T compound; Tissue-Tek, Sakura Finetechnical Co., Ltd., Tokyo, Japan), frozen at −80 °C, and stored until sectioning. Similarly, plantar flexor muscles (PLA, SOL, GAS) were freshly removed and weighed to evaluate the atrophy rate of muscles. Four mice in the CT and seven mice in the NT groups were directly used for the histology without the functional examination. 

Subsequently, to examine the numerical recovery of the damaged sciatic nerve, several 7 µm cross-sections were obtained from four portions, as shown in Figure 1A. Position 1 was a cross-section showing the proximal portion of the conduit, and positions 2–4 were sections in the conduit showing nerve regeneration profiles. Engrafted GFP was detected by fluorescence stereomicroscopy (Figure 1B). Localization of nerve fibers (axons) was determined by rabbit polyclonal anti-neurofilament 200 (N200, 1:1000, room temperature for 1 h; Sigma, Saint Louis, MO, USA). Myelin formation was detected by rabbit polyclonal anti-myelin basic protein (MBP; 1:200, room temperature for 2 h; Millipore, Billerica, MA, USA). Reactions were visualized using Alexa Fluor-594-conjugated goat anti-rabbit and anti-rat antibodies (1:500, room temperature for 2 h; Molecular Probes, Eugene, OR, USA. Nuclei were counter-stained with 4,6-diamidino-2-phenylindole (DAPI). Histological photographs were taken with a fluorescence multi focal projection system using Stereo Investigator (mbf Bioscience, MicroBrightField Inc., Williston, VT, USA) and fluorescence microscopy (Olympus BX61 with U-HGLGPS and BX-UCB, Tokyo, Japan). Histological numerical analyses (axons and myelin) were performed as indicators of numerical recovery of the damaged sciatic nerve. All axons and myelinated fibers were detected in the whole sciatic nerve cross-sections to assess absolute numerical recovery. In this respect, we used the mbf tile image method with higher magnification photographs.

### 2.6. Protein Analysis for the Cytokine Cocktail

To elucidate the components of the cytokine cocktail, cyclopedic protein analysis was performed using an antibody array kit (Proteome Profiler Mouse Angiogenesis and Adipokine Array Kit, ARY015 and ARY013, R&D Systems Inc., Minneapolis, MN, USA). With these two systems, 78 proteins were analyzed (a total of 91; however, 13 were overlapped). Cell culture supernatants of Sk-MSCs, prepared as the cytokine cocktail (500 µL), were also prepared for protein analysis. IMDM containing 20% AMS, which was used for NT group administration, was also used for the control and the relative protein expression levels of selected cytokines were determined. In this analysis, it was not clear how many nerve regeneration-related factors in this angiogenesis and adipokine-related proteome arrays were considered. Therefore, we also analyzed the normal and damaged sciatic nerve lysates and compared the results. Hence, the nerve crush injury model [8] was used for the right sciatic nerve of the wild-type mouse (*n* = 6) under anesthesia. Five days after the crush injury, sciatic nerves on both sides were removed and prepared for abstraction of the normal nerve lysate (left side) and the damaged nerve lysate (right side). The sciatic nerve crush injury model was used as a confirmation model to determine which cytokine was closely linked to nerve regeneration in this analysis. All samples were normalized by protein concentration, and applied appropriate amount following the instruction of the array kit. Relative expression of each samples was expressed by the pixel intensity based on the reference spot. 

### 2.7. Statistical Analysis

Differences between the functional and numerical data of the two groups (CT- and NT-group) were tested using the Student’s *t*-test (for the absolute values) and Wilcoxon non-parametric test (for the percentage value), and the significance level was set at *p* < 0.05. Values are expressed as the mean ± SE.

## 3. Results

### 3.1. Final Body and Muscle Mass, and Tension Output of Downstream Plantar Flexor Muscles

Final body and muscle masses in both groups are summarized in Table 1. The final body mass and muscle mass of the contralateral side in both groups were quite similar; thus, it was considered that feed and drink with their health-related factors, such as the food, water, temperature, humidity, and administration conditions, were the same in both groups during the experiment. Under these conditions, the CT group showed a relatively higher absolute muscle mass (although not significant). 

Alternatively, when comparing the tetanic tension output of plantar flexors, the CT group showed significantly higher values than the NT group, whereas the value of the contralateral side was similar. This suggests that more recruitment of motor units occurred in plantar flexor muscles in the CT group according to the upstream nerve re-innervation/re-connection.

**Figure 2:**
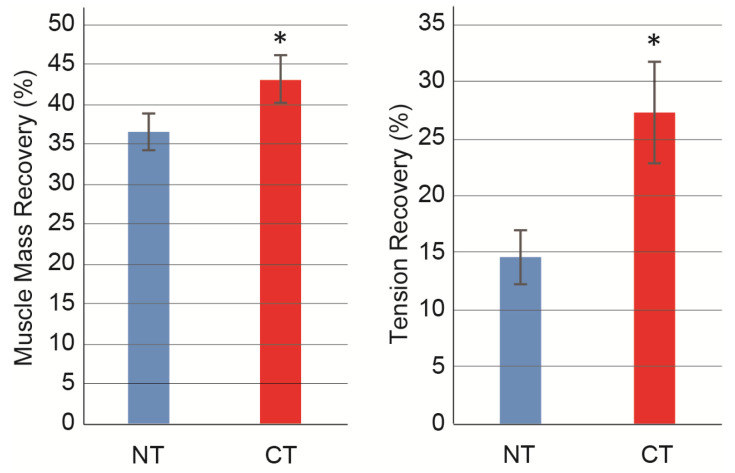
Recovery ratio of muscle mass and tetanic tension output. Values are expressed as mean percentages for the non-operated contralateral side in each individual. Muscle mass and tetanic tension output were evaluated as total mass and tension output of planter flexor (plantaris + soleus + gastrocnemius) muscles. Muscle mass and tension recovery ratio was calculated as follows; (operated muscles mass/contralateral muscles mass) × 100, (tension output of operated muscles/tension output of contralateral muscles) × 100. NT, non-cytokine administered control group; CT, cytokine administered group. Values are expressed as mean ± SE. * *p* < 0.05.

Significant differences between the NT and CT groups were further defined in the recovery ratio of the muscle mass and tetanic tension of plantar flexor muscles based on the contralateral side (Figure 2). The CT group showed significantly higher values for both muscle mass and functional recovery. 

### 3.2. Morphological and Numerical Recoveries 

**Figure 3:**
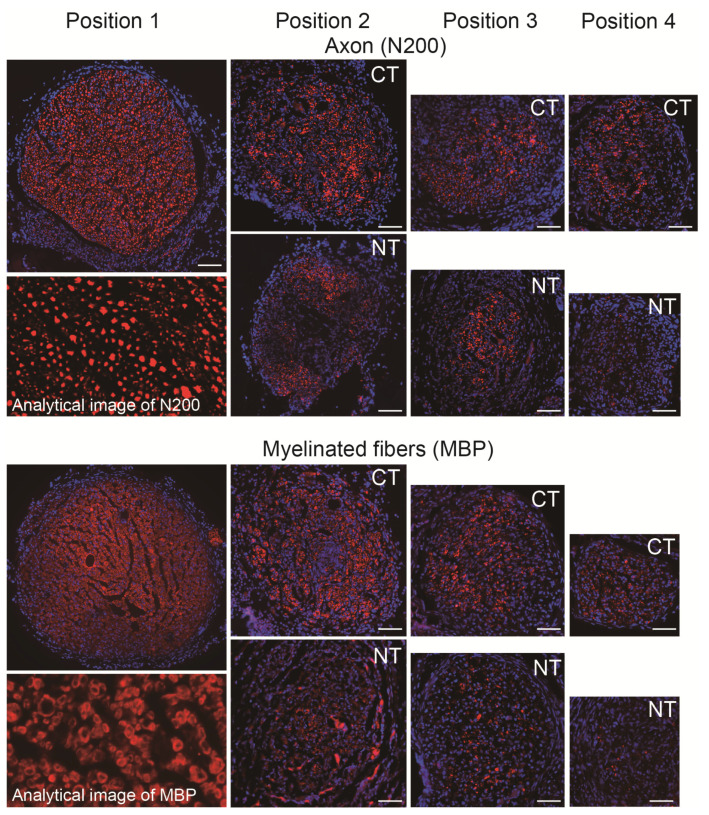
Typical photographs of axon and myelinated fiber formation in regenerated sciatic nerves in the CT and NT groups. Position 1 indicates upstream of the damaged site, thus common in both groups (each position refers to Figure 1). Sections were stained with N200 and MBP (red) as axon and myelin staining, and nuclear staining with DAPI (blue). Photographs were composed by the tiling image method to present whole features of the sciatic nerve. The analytical image displays the typical characteristics of quantitative analysis. Scale bars = 100 μm. NT, non-cytokine control group; CT, cytokine group; MBP, anti-myelin basic protein; DAPI, 4′,6-diamidino-2-phenylindole.

Typical photographs of the operated nerve fibers in the CT and NT nerves, such as the formation of axons (N200) and myelin (MBP), are presented in Figure 3. Six weeks after the operation, both groups showed a reduced number of axons and myelinated fibers in damaged positions 2–4 compared with the upstream non-damaged portion (position 1). However, the CT nerve showed a higher density of axons and myelinated fibers than the NT nerve in each position. 

**Figure 4:**
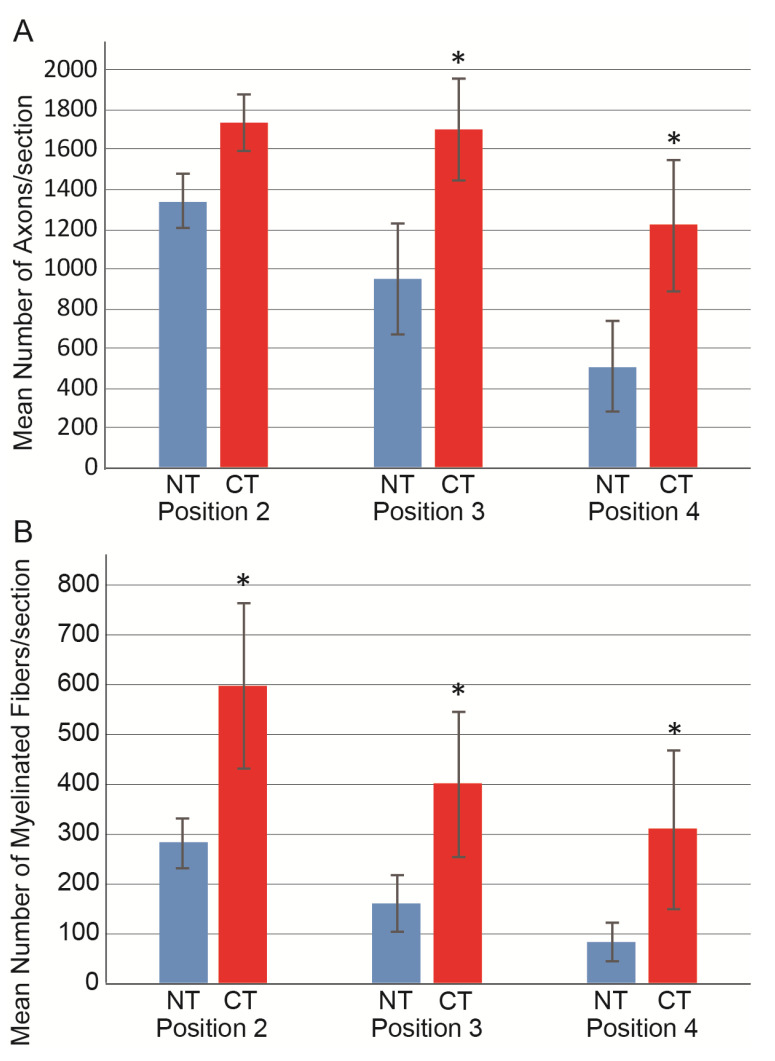
Number of axons and myelinated fibers in the CT (*n* = 6) and NT (*n* = 10) groups. (**A**) Mean number of axons/section. (**B**) Mean number of myelinated fibers/section. Values were obtained from three positions in the damaged sciatic nerve (positions 2–4, refer to Figure 1) and averaged. Values are presented as the mean ± S.E. * *p* < 0.05 (CT vs. NT). NT, non-cytokine administered control group; CT, cytokine administered group.

The above morphological analysis was quantified and presented as the numerical recovery of axons and myelinated fibers in damaged portions, as shown in Figure 4. For axon counts at positions 2–4, consistently higher values were observed in the CT group than in the NT group, with significant differences observed in positions 3 and 4 (Figure 4A). A similar trend was also observed in the count of myelinated fibers, and significant differences were detected in all three positions (Figure 4B). 

**Figure 5:**
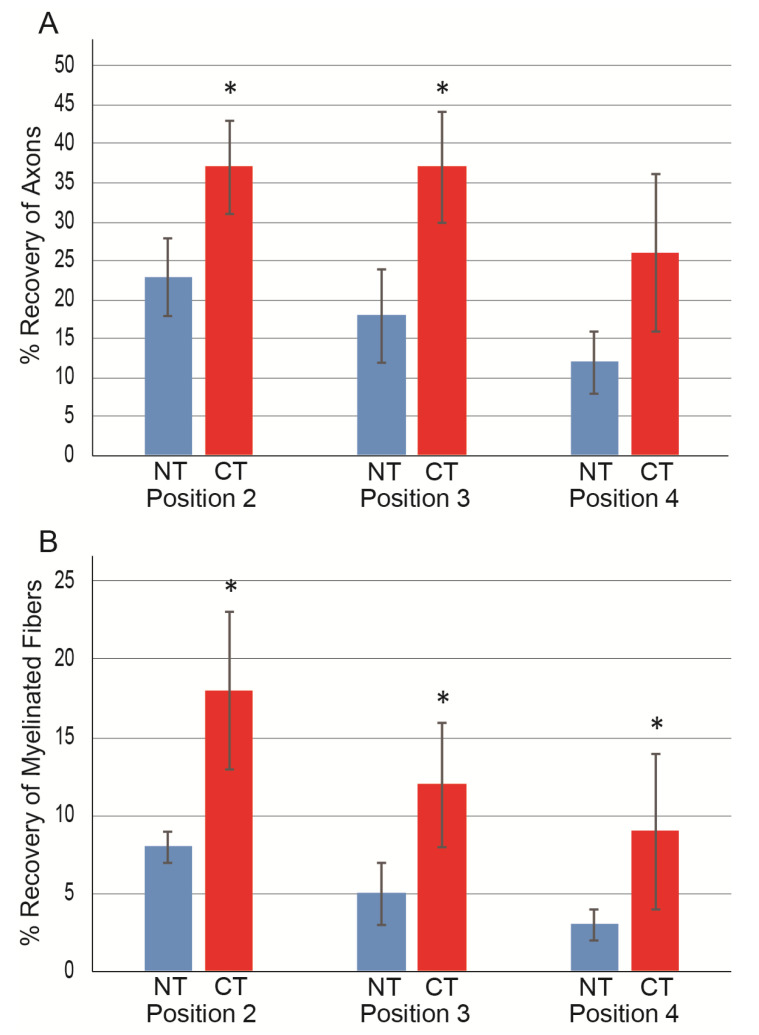
Recovery ratio of axons and myelinated fibers in each position in the CT (*n* = 6) and NT (*n* = 10) groups. Data were calculated based on pooled values of non-damaged upstream position 1 in both groups (*n* = 20) as N200; 4625 ± 142, MBP; 3329 ± 114. (**A**) Axon recovery ratio; (**B**) myelinated fiber recovery. Individual values are presented as the mean ± S.E. * *p* < 0.05 (CT vs. NT). NT, non-cytokine control group; CT, cytokine group.

The recovery ratios of axons and myelinated fibers in each position are presented in Figure 5 and are based on the pooled data of position 1. The CT group consistently showed significantly higher recovery ratios in all positions (*p* < 0.05), except for position 4 in the axon. An almost two-fold recovery ratio was obtained in the CT group compared with the NT group. 

### 3.3. Factors of the Cytokine Cocktail

**Figure 6:**
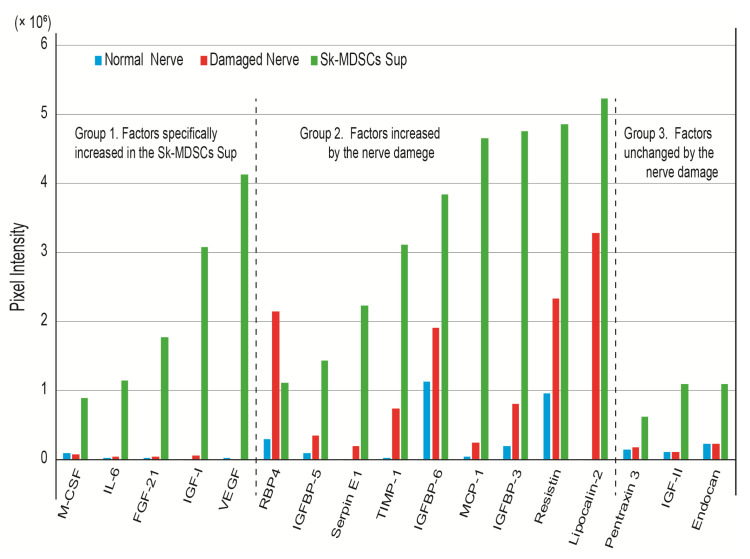
The main factors of the cytokine cocktail. These were determined by comparison of Group 1: factors specifically increased/detected in the Sk-MSC supernatant and almost undetected in both the normal and damaged nerve; Group 2: factors increased in the damaged nerve (minimally 1.5-fold greater in normal vs. damaged); and Group 3: factors unchanged by nerve damage but increased in Sk-MSCs. Significant change of values for the baseline was determined using >0.1-pixel intensity. Sk-MSCs, skeletal muscle-derived stem cells.

To determine the main component of the cytokine cocktail, cyclopedic protein analysis was performed using an antibody array kit. The main factors of the cytokine cocktail are shown in Figure 6. In addition, to directly elucidate nerve regeneration-related factors, the supernatant of the Sk-MSCs (cytokine cocktail) was compared to the extracts of normal and damaged nerves as Group 1, 2, and 3. 

From the above refinement, the factors were narrowed down to 17 against 78. Group 1 factors were considered Sk-MSC-specific cytokines with no relationship to nerve tissue in both normal and damaged states. These included macrophage colony stimulating factor (M-CSF), IL-6 (interleukin-6), FGF-21 (fibroblast growth factor 21), IGF-1 (insulin-like growth factor-1), and vascular endothelial growth factor (VEGF). Group 2 factors were considered nerve damage-related factors, and these included RBP4 (retinol binding protein 4), IGFBP-5 (insulin-like growth factor-binding protein 5), Serpin E1, TIMP-1 (metallopeptidase inhibitor 1), IGFBP-6, MCP-1 (monocyte chemoattractant protein-1), IGFBP-3, Resistin, and Lipocalin-2. Notably, all these factors except RBP4 were also detected in Sk-MSCs. Group 3 factors were detected in nerve tissue; however, these were unchanged by the damage, and increased in Sk-MSCs: Pentraxin 3, IGF-II (insulin-like growth factor-2), and Endocan. The amplitude of the increasing ratio in Sk-MSCs was relatively low compared to the other two groups. 

## 4. Discussion

In the present study, the CT group clearly indicated a significant facilitation effect of sciatic nerve regeneration compared with the NT group, both numerically and functionally. Therefore, it was proven that administration of the Sk-MSC culture supernatant (cytokine cocktail) is undeniably effective in peripheral nerve regeneration in vivo. To the best of our knowledge, this is the first study to quantify the relationship between nerve regeneration and cytokine cocktail treatment from an in vivo therapeutic viewpoint. Functional and numerical recovery ratios of the present combination therapy using scaffold tube bridging and cytokine cocktail administration was approximately 20–30%, which is lower than a previous study where tube bridging and Sk-MSC transplantation was used (80–90% recovery) [8]. Therefore, this may be insufficient for nerve injury therapy with a large deficiency (long gap). However, from another viewpoint, this has a two-fold greater recovery than scaffold bridging treatment (NT group) alone, and this facilitation rate may be sufficient in clinical practice. In addition, this is regenerative medicine without the use of stem cell transplantation, however still using stem cell functions. Hence, this would make clinical application much easier than cell transplantation therapy in every country. Therefore, this treatment may be clinically available for recovery of severe (intermediate degree) nerve injury without gap transection, induced by penetrations, crushes, tractions, and lacerations, via needle injection, once a week for several weeks. Note that we think that the sole use of this cocktail is insufficient to the therapy for the seriously severe nerve injury with long gaps, such as the present model. For such a serious nerve injury, direct Sk-MSC cell transplantation should be required [8,11].

In the present study, we used the GFP nerve graft in the center of the collagen tube consistently in the NT and CT group, as an indicator to correctly obtain histology sections 1 to 4 of Figure 1A. In regard to the GFP nerve graft, we reported that the GFP graft limitedly contributed as the Schwann cell supplier, but the effect of axon and myelinated fiber growth was small and not significantly different from that of the media group [8]. Therefore, we think that this is not the main factor of the present study. 

Additionally, we selected six weeks of recovery time in the present study. Based on our previous study [11], it was strongly suggested that the paracrine effects of Sk-MSCs was most prominent during first 2–3 weeks of post-treatment. Then, this effect declined towards four weeks, and the effect remained almost the same level at eight weeks thereafter. Therefore, we selected six weeks as the sufficient and appropriate period to evaluate the cytokine ability. In this regard, it is supposed that the first 2–3 weeks of post-nerve injury may be the most effective and important phase of this cytokine cocktail treatment. Therefore, this treatment should be started at the acute phase and may be too late in the subacute phase. 

Considering the clinical application of this method, autologous sample accession of skeletal muscle is not problematic because human Sk-MSCs can be obtained from the rectus abdominis muscle [19]. Obtaining a small sample of skeletal muscle (5–10 g) from the rectus abdominis may be less invasive or similar to bone marrow acquisition and is definitely less invasive than an appendectomy because it avoids contact with the internal organs. In addition, freeze storage is possible for the extracted cytokine cocktail, and expanded human Sk-34 and Sk-DN/29+ cells (corresponding present Sk-MSCs) can also be stored by freezing and can be re-used after thawing [11,19]. 

Similar conception therapy has been performed as platelet-rich plasma (PRP) treatment [20,21]. PRP is an autologous concentration of human platelets at supra-physiologic levels, and includes platelet-derived growth factor, epidermal growth factor, transforming growth factor beta 1, VEGF, basic FGF, hepatocyte growth factor, and IGF-I in its alpha granules, as natural reservoirs [22,23]. Several preclinical and clinical studies have demonstrated the beneficial effects of PRP therapy, particularly in oral and maxillofacial surgery (such as bone reconstruction prior to dental implant) [24,25], dermatology, and plastic surgery (such as skin wound healing and scar revision, skin rejuvenating effects following stimulation of dermal fibroblast proliferation and increases in type I collagen synthesis, soft-tissue augmentation, fibroblast activation, new collagen deposition, new blood vessels, and adipose tissue formation) [12,23,26], and orthopedic surgery and sports medicine (such as acute hamstring injuries, Achilles and patellar tendinopathy, osteoarthritis, chondrocyte and synoviocyte stimulation, rotator cuff tear, and injuries and pathologies associated with ligaments, tendons, muscles, cartilage, and bone) [21,27,28,29,30,31]. Although a large number of reports exists, there is still room for debate on the effectiveness of PRP treatment. It is also likely that the adverse effects are rarely reported, showing that this kind of injection therapy is relatively safe. 

The mechanism underlying the beneficial effects of PRP administration is unclear. However, it is also hypothesized that most of the beneficial effects of PRP treatment are dependent on angiogenesis and cellular activation of reference tissues around the injected portion, induced by the concentrated cytokines above. 

Therefore, we determined 17 therapeutic candidates for factors in the present study, including VEGF, FGF, and IGF-I at the protein level. The molecular pathway of these cytokines was already published, and they were mostly related to the angiogenesis, because of the basis of the protein array kit (angiogenesis). Thus, it was supposed that there were close relation mechanisms of molecular events between nerve regeneration and angiogenesis. However, further study is needed to clarify the direct relationship to the nerve regeneration. The expression of other specific nerve growth and neurotrophic factor mRNAs in Sk-MSC, such as nerve growth factor, brain-derived neurotrophic factor, glial-derived neurotrophic factor, Galectin, Ninjurin, ciliary neurotrophic factor, and leukemia inhibitory factor were reported before (in culture) and after Sk-MSC transplantation (in vivo) in a previous study [8]. Furthermore, the present cytokine cocktail also included a sufficient volume of factors that were increased by nerve damage (Group 2; probably inevitable for the nerve regeneration process). Therefore, it was suggested that the present nerve regeneration facilitation effect was induced by cross-interaction and/or combined effects of these factors. These could affect and/or activate axonal growth of transected nerve stumps and with the nerve regeneration-related surrounding cells, such as Schwann cells and perineural/endoneurial cells. Cells linked to angiogenesis/vasculogenesis, such as endothelial cells, pericyte and vascular smooth muscle cells, were also affected for their proliferation/differentiation. 

Furthermore, in previous studies, Sk-MSCs consistently exerted the synchronized tissue reconstitution ability of the nerve–muscle–blood vessel unit in the skeletal muscle [18,32,33], muscle–tendon unit [34], and in a variety of other tissues, i.e., around the urinary tract [35,36,37], bladder wall [38], bronchial stump [39], facial nerve [40], and recurrent laryngeal nerve [41]. Therefore, it is also possible that this cytokine cocktail treatment may be available for a variety of tissue reconstitution therapies as a tissue regeneration facilitation agent. 

## 5. Conclusions

This study demonstrated the first step of regenerative medicine without using stem cells; however, still employing its functions, aiming for a broader and easier adjuvant therapeutic utility with the cytokine cocktail derived from Sk-MSCs. This cocktail showed facilitating ability for the peripheral nerve regeneration based on the recovery of the reconnected number of axon and myelinated fibers and the tetanic tension output via electrical stimulation. This cocktail typically contained several angiogenesis and peripheral nerve regeneration relating factors; thus, it was possibly expected to the facilitate the nerve–vascular regeneration in the variety of tissues. Further studies to elucidate other specific factors expressed by Sk-MSCs and development of a condensed method using this cytokine cocktail are necessary. 

## Figures and Tables

**Table 1:** Body and muscle mass, and muscle tension output.

Group	Body Mass (g)	Muscle Mass (mg)		Tetanus (N 10^2^)	
		PLT + GAS + SOL		PLT + GAS + SOL	
		Cont-side	Op-side	Cont-side	Op-side
NT (*n* = 8)	20.8 ± 1.7	139 ± 9	51 ± 5	103.4 ± 7.7	15.2 ± 2.7
CT (*n* = 6)	20.8 ± 1.5	138 ± 9	57 ± 4	105.1 ± 4.8	* 27.8 ± 4.7

NT, non-cytokine administered control group; CT, cytokine administered group; Cont-side, control- side; Op-side, operation side. N, Newton. PLT, Plantaris; GAS, Gastrocnemius; SOL, Soleus. Values are expressed as mean ± SE. * *p* < 0.05.

## Data Availability

The data presented in this study are available on request from the corresponding author.
